# COVID-19-Stresstest für die Sicherstellung der Notfallversorgung: Strategie und Maßnahmen der Notfallrettung in Berlin

**DOI:** 10.1007/s00101-020-00890-8

**Published:** 2020-11-27

**Authors:** Janosch Dahmen, Linnart Bäker, Florian Breuer, Karsten Homrighausen, Christopher Pommerenke, Jan-Karl Stiepak, Stefan Poloczek

**Affiliations:** 1 Berliner Feuerwehr, Berlin, Deutschland; 2 Ärztliche Leitung Rettungsdienst im Land Berlin, Berlin, Deutschland; 3 Fakultät für Gesundheit, Department Humanmedizin, Universität Witten/Herdecke, Alfred-Herrhausen-Straße 50, 58448 Witten, Deutschland

**Keywords:** Berliner Feuerwehr, Pandemie, Rettungsdienst, Corona, SARS-CoV‑2, Berlin Fire Brigade, Pandemic, Emergency medical services, Corona, SARS-CoV‑2

## Abstract

**Hintergrund:**

Die COVID-19-Pandemie stellt die Notfallversorgung in Deutschland vor eine beispiellose Belastungsprobe. Neben der klinischen Notfallversorgung in den Notaufnahmen der Krankenhäuser kommt der präklinischen Notfallrettung die entscheidende Sicherstellungsaufgabe notfallmedizinischer Gesundheitsversorgung zu. Die Berliner Feuerwehr zeigt in dem vorliegenden Beitrag für die Notfallrettung im Land Berlin neue Wege auf, dieser gewachsenen Verantwortung in der prähospitalen Patientenversorgung gerecht zu werden.

**Methode:**

Es erfolgte eine systematische Darstellung der Herausforderungen und konzeptionellen Antworten der präklinischen Notfallmedizin auf die COVID-19-Pandemie am Beispiel der Notfallrettung im Land Berlin.

**Ergebnisse:**

Die Berliner Feuerwehr koordiniert in einer zentralen Leitstelle für das Land Berlin alle Hilfeersuchen des Notrufs 112. Je 24 h gehen im Mittel 2565 Notrufe ein, aus denen 1271 Einsätze generiert werden. Im Rahmen der Pandemie kam es zu einer deutlichen Zunahme an Einsätzen zu Patienten mit akuten respiratorischen Erkrankungen (ARE). So erfolgten 11 % der Einsätze zu Patienten mit dem Verdacht einer COVID-19-Erkrankung. Die Notrufdauer verlängerte sich bei ergänzender Abfrage des „Pandemie-Protokolls“ im Schnitt um 1:36 min, die Einsatzdauer bei Einsätzen mit Alarmstichwort-Zusatz „akute respiratorische Erkrankung [ARE.]“ im Mittel um 17 min.

**Schlussfolgerung:**

Die andauernde Pandemie macht deutlich, dass Aufgaben und Verantwortung der öffentlichen Träger der Notfallrettung über die unmittelbare, medizinische Gefahrenabwehr für Leib und Leben hinausgehen. Neben einer Lotsen- und Triage-Funktion in der integrierten Leitstelle der Berliner Feuerwehr (112) konnte durch umfassende Maßnahmen der Lageüberwachung, Lagedarstellung und Lagebewältigung trotz Ausfall alternativer, ambulanter Versorgungsangebote u. a. im Bereich der Haus- und Facharztpraxen die Notfall- resp. Gesundheitsversorgung sichergestellt werden.

**Zusatzmaterial online:**

Die Online-Version dieses Beitrags (10.1007/s00101-020-00890-8) enthält weiteres Online-Zusatzmaterial.

Beitrag und Zusatzmaterial stehen Ihnen auf www.springermedizin.de zur Verfügung. Bitte geben Sie dort den Beitragstitel in die Suche ein, das Zusatzmaterial finden Sie beim Beitrag unter „Ergänzende Inhalte“.

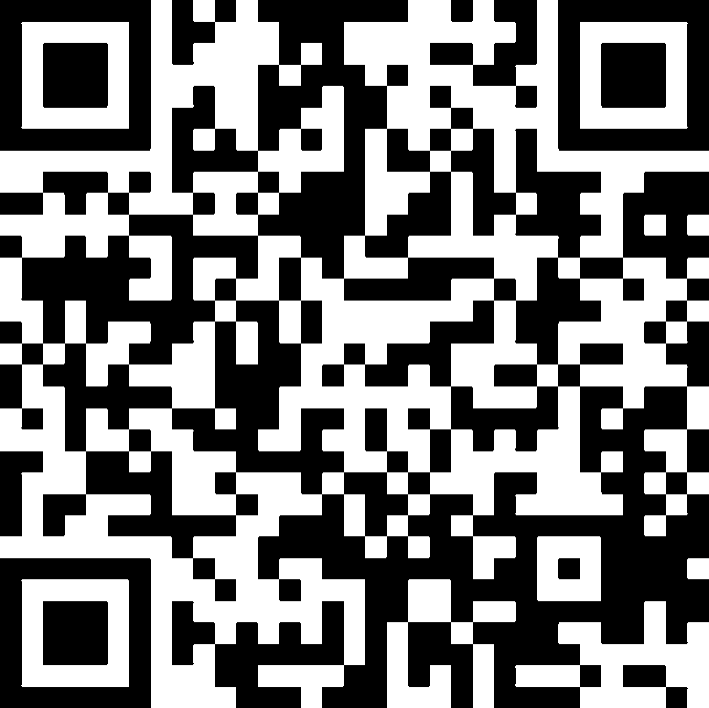

## Abstract (en)

**Background:**

The COVID-19 pandemic represents an unprecedented severe test for emergency medicine in Germany. In addition to in-hospital emergency medicine, prehospital emergency medicine has the decisive task of fully guaranteeing emergency medical healthcare. In this article the Berlin Fire Brigade shows new ways for emergency medical services to fulfil these increased responsibilities during the pandemic in prehospital emergency medicine in the State of Berlin.

**Methods:**

A systematic presentation of the challenges and conceptional responses of preclinical emergency medicine to the COVID-19 pandemic was carried out using the example of the emergency medical services in the State of Berlin.

**Results:**

The Berlin Fire Brigade has a dispatch center that coordinates all requests for assistance in the State of Berlin over the emergency telephone number 112. On average a total of 2565 emergency calls are received every 24 h, from which 1271 missions are generated. During the pandemic there was a striking increase in missions to patients with acute respiratory diseases (ARD). Of the missions 11% were carried out to patients with the suspicion of COVID-19. The duration of the emergency calls was extended on average by 1:36 min due to the additional questions in the pandemic protocol and the duration of the mission by an average of 17 min with the additional alarm keyword acute respiratory disease (ARD).

**Conclusion:**

The continuing pandemic reveals that tasks and responsibilities of public services in emergency rescue go far beyond the immediate medical prevention of danger to life and limb. In addition to the controller and triage functions in the integrated dispatch center of the Berlin Fire Brigade (112), the emergency and healthcare measures could be ensured. This was accomplished by comprehensive measures for situation control, situation reports and mastering situations despite the lack of alternative outpatient care options, especially in the areas of general practitioner, public health care and medical specialist practices.

## Ausgangslage

Am 05.01.2020 wurde die Ärztliche Leitung des Rettungsdienstes der Berliner Feuerwehr im Land Berlin (ÄLRD) über das Frühwarnsystem des European Emergency Medical Services Leadership Network (EMS Leadership Network) [1] und die WHO auf eine neuartige, infektiöse Lungenerkrankung in der Stadt Wuhan aufmerksam [2]. Am selben Tag berichtete die internationale Presse erstmals über 59 bekannte Infektionsfälle, davon 11 kritisch erkrankte Menschen mit einer bilateralen, atypischen Pneumonie in der genannten zentralasiatischen Region Hubei [3]. In den ersten Berichten gingen die verantwortlichen Stellen der Wuhan Municipal Health Commission zu diesem Zeitpunkt noch davon aus, dass eine Mensch-zu-Mensch-Übertragung unwahrscheinlich sei, die erste Infektion sich voraussichtlich am 12.12.2019 zugetragen habe und bisher keine Ansteckungen unter dem Personal des Gesundheitswesens in der Region festzustellen worden sei.

Vor dem Hintergrund der Erfahrungen des epidemiologischen Managements des „severe acute respiratory syndrome“ (SARS) 2002/2003 und des „middle east respiratory syndrome“ (MERS) 2012 wurden mit Bekanntwerden der Entwicklungen durch die ÄLRD als erste Maßnahmen die enge Beobachtung der epidemiologischen Entwicklung, insbesondere respiratorischer Erkrankungen, und die spezifische kontinuierliche Abstimmung mit den Partnern des EMS Leadership Network veranlasst.

Aufgrund der schnellen Ausbreitung der Infektion in den folgenden Wochen wurde am 20.01.2020 eine erste Surveillance-Arbeitsgruppe unter Leitung der Oberärztin bzw. des Oberarztes vom Dienst (OAvD) zur Identifikation notwendiger Handlungsfelder im Umgang mit COVID-19-Patienten durch den ÄLRD initiiert.

Durch die Arbeitsgruppe wurde neben der täglichen Lagedarstellung auch am 24.01.2020 eine erste „standard operating procedure“ (SOP) für den Umgang mit potenziell infizierten Patientinnen und Patienten im Rettungsdienst veröffentlicht. Am 28.01.2020 wurde dann die erste Infektion in Deutschland in Bayern bei einem Patienten nachgewiesen [4]. Es folgte eine sehr dynamische und noch andauernde epidemiologisch bedeutsame Lage, die als andauernder Einsatz das gesamte Gesundheitswesen und insbesondere auch die Notfallrettung bis zum heutigen Tag herausfordert.

Der Artikel gibt vor dem Hintergrund der derzeit diskutierten Reform der Notfallversorgung in Deutschland einen Überblick über die Strategie und die bislang umgesetzten Maßnahmen der Berliner Feuerwehr und der Ärztlichen Leitung Rettungsdienst im Land Berlin im Umgang mit der andauernden SARS-CoV-2-Pandemie. Aus den dargestellten Erkenntnissen werden die Bedeutungen eines umfassenden Sicherstellungsbegriffs, der klaren Zuständigkeit in der medizinischen Gefahrenabwehr resp. Notfallversorgung und auch von sektorenübergreifenden Versorgungskonzepten vor dem Hintergrund der genannten Gesetzesreform deutlich.

## Aufbau eines Pandemiestabs

Zur Bewältigung der ab Anfang Februar sehr dynamisch anwachsenden Pandemielage wurde durch die Behördenleitung der Berliner Feuerwehr der Einsatzplan für „epidemisch bedeutsame Lagen“ aktiviert und ein Pandemiestab mit rund 15 festen Funktionen eingerichtet. Der Stab wurde im 4‑Schicht-System mit getrennten Kohorten und kontaktlosen Übergaben organisiert. Hierbei erfolgte die Übergabe mittels einer Videokonferenz; weiterhin waren Eingang und Ausgang zum Stabsraum getrennt voneinander, und die jeweiligen Stabsgruppen konnten sich aufgrund eines Zeitversatzes nicht im Stabsraum begegnen. Zur Verstärkung wurden im Verlauf in einem erweiterten Stabszellenkonzept Organisationseinheiten der Linienorganisation der Berliner Feuerwehr zu sog. 24/7-Stabszellen ertüchtigt und eine dezentrale Rund-um-die-Uhr-Verfügbarkeit aller kritischen Arbeitsprozesse insbesondere auch jenseits des Einsatzgeschehens hergestellt. Außerhalb des Einsatzbetriebes, des Pandemiestabs und der angegliederten Stabszellen wurde sämtliche rückwärtige Arbeit über digitale Hilfsmittel überwiegend auf Heimarbeitsplätze verlagert.

## Überwachung des Einsatzgeschehens und Darstellung besonderer Einsatzschwerpunkte

Über den gesamten Zeitraum, bereits von der Frühphase der epidemiologischen Entwicklung ausgehend, stellte das Einsatzgeschehen letztlich indirekt ein immanentes, infektiologisches Frühwarnsystem durch qualitative und quantitative Veränderungen im Anrufverhalten und im Einsatzgeschehen da. Die Berliner Feuerwehr koordiniert in einer zentralen Leitstelle für das Land Berlin alle Hilfeersuchen, die über den Notruf 112 eingehen. Im Jahresmittel gehen hier je 24 h 2565 Notrufe ein, aus denen im Mittel 1271 Einsätze generiert werden. In den Monaten März/April kam es wiederholt zu über 4000 Notrufen (im Mittel 3523) und bis zum Beginn des sog. Lockdowns auch zu einer deutlichen Zunahme von Einsätzen, bei zeitgleicher Zunahme der Einsatzdauer in Teilen des Einsatzgeschehens (Tab. 1).

**Table 1:** 

Alarmstichwort	Mit/ohne ARE.	Einsatzdauer in min (Mean)	SD	Einsatzdauer in min (Median)
*NA*	Mit ARE	86,4	±39,8	76,2
*NA*	Ohne ARE	72,6	±35,4	69,4
*NOTF*	Mit ARE	69,2	±28,1	65,9
*NOTF*	Ohne ARE	58,8	±26,9	56,4
*NT/NT D*	Mit ARE	64,9	±28,3	60,6
*NT/NT D*	Ohne ARE	62,3	±34,1	57,3
*Sonstige*	Mit ARE	88,6	±51,4	80,8
*Sonstige*	Ohne ARE	47	±46,3	38

*NA* Notarzt, *NOTF* Notfall, *NT/NT D* Notfalltransport/Dringlich

Sämtliche Einsätze in der Leitstelle werden unter Anwendung einer standardisierten Notrufabfrage abgefragt. Strukturierte oder standardisierte Notrufabfragesysteme sind in Deutschland bislang nicht flächendeckend etabliert [5]; die Verwendung international ist deutlich höher.

Im Ergebnis der standardisierten Notrufabfrage wird in Abhängigkeit des ermittelten Leitsymptoms oder der geschilderten Beschwerden einer von 4608 „dispatch codes“ generiert, an die wiederum spezifische Reaktionen in der Alarm- und Ausrückeordnung angebunden sind. In der Frühphase der Pandemie wurde am 26.02.2020 mit dem Ziel der Surveillance und Triage das Epidemieprotokoll des Advanced Medical Priority Dispatch System (AMPDS Protokoll 36 Pandemie/Epidemie/Ausbruch) aktiviert [6, 7]. Somit bestanden die Möglichkeiten der Surveillance des Ausbruchsgeschehens, der besseren Einsatzsteuerung und insbesondere Sensibilisierung der Einsatzkräfte, einen Alarmstichwort-Zusatz „[ARE.]“ für akute respiratorische Erkrankung an das Einsatzstichwort anzubinden.

Bis zum 01.09.2020 wurden insgesamt 17.057 Einsätze mit dem Protokoll 36 abgefragt. Im Schnitt dauerten die standardisierte Notrufabfrage mit Einstiegs- und Schlüsselfragen über alle Hauptbeschwerden 1:10 min, die Abfrage des Pandemieprotokolls 36 durchschnittlich zusätzlich 1:36 min. Seit Beginn der Pandemie sind bis September 2020 so allein 386 h zusätzliche Notrufabfragezeit angefallen.

**Figure 1:**
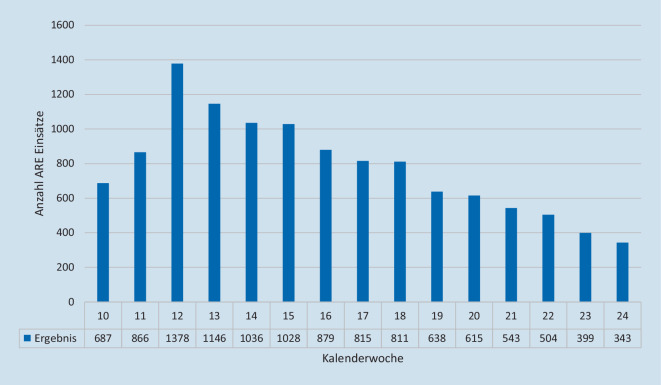

Bei 11 % der Gesamteinsätze wurden im Zeitraum März–Juni 2020 Einsatzmittel zu hilfsbedürftigen Menschen mit Verdacht auf eine akute respiratorische Erkrankung (Abb. 1) geschickt. Die Einsätze mit einem Alarmstichwort-Zusatz [ARE.] dauerten im Schnitt über alle Alarmstichworte im Mittel (Median) 17 min länger als Einsätze ohne Alarmstichwort-Zusatz [ARE.] (Tab. 1), wobei Einsätze mit einem Notarzt (NA + ARE) im Durchschnitt +14 min länger dauerten als alle übrigen Einsätze eines Notarztes, gefolgt von Einsätzen eines RTW zu einem Notfall (NOTF + ARE) +10 min im Vergleich zu gleichen Einsätzen ohne ARE-Zusatz. Der geringste Unterschied zeigte sich bei Notfalltransporten (NT + ARE) mit einer durchschnittlich zusätzlichen Dauer +3 min. Allein im Monat April 2020 fielen bei gleichen Alarmstichworten insgesamt zusätzlich über 900 h der verlängerten Einsatzdauer bei ARE-Einsätzen an.

Bei jedem Einsatz zu einem ARE-Alarmstichwort erfolgte von der Einsatzstelle anschließend eine standardisierte Kurzlagemeldung durch die Einsatzkräfte in Analogie zur RKI-Falldefinition (Zusatzmaterial online: „Falldefinitionen und Kurzlagemeldungen in Analogie zur RKI-Falldefinition“; s. Hinweis am Anfang des Artikels).

Die Anmeldung von ARE-Patienten im Krankenhaus erfolgte für alle Einsätze zentral über die Plattform für den interdisziplinären Versorgungsnachweis https://www.ivena.de.

Insbesondere zu Beginn der epidemiologischen Entwicklungen wurde die Leitstelle mit einer überdurchschnittlich großen Anzahl reiner Informationsersuchen konfrontiert, bei denen kein medizinisches Hilfeersuchen in Anlehnung an das Rettungsdienstgesetz Berlin mit originärer Zuständigkeit der Berliner Notfallrettung erkennbar war. Im Kern drehte sich der überwältigende Teil dieser Anrufe um Fragen zu Testmöglichkeiten und der Erreichbarkeit des Gesundheitsamts sowie von Hausärzten. Nach Einrichtung einer landesweiten COVID-19-Informationshotline der Senatsverwaltung für Gesundheit, Pflege und Gleichstellung wurden deshalb die Mitarbeitenden der Leitstelle für eine schnelle Differenzierung zwischen Informations- und Hilfeersuchen sensibilisiert und wurden zur Weiterleitung reiner Informationsersuchen an die COVID-19-Informationshotline befähigt.

Zur Verstärkung im Aufgabengebiet der Notrufannahme wurden in einem 14-tägigen Kurs 23 Auszubildende zum Notfallsanitäter der Berliner Feuerwehr und Rettungsdienst Akademie (BFRA) unter enger Aufsicht des Qualitätsmanagements der Leitstelle qualifiziert und so die Leitstelle von 25 Funktionen (Einsatzkräften) im Tagdienst auf 31 Funktionen aufgestockt. Darüber hinaus wurde die bereits vor der Lage implementierte elektronische Schnittstelle zum bidirektionalen, digitalen Austausch von Hilfeersuchen der Leitstelle zum Ärztlichen Bereitschaftsdienst der kassenärztlichen Vereinigung (KV) ausgeweitet, um nach standardisierter Notrufabfrage eine Weitergabe von Anrufen zu ermöglichen. Seit der Aktivierung des Protokolls 36 wurden so 7397 niedrigprioritäre medizinische Hilfeersuchen der 112 an die Leitstelle der KV übermittelt, in Spitzenzeiten bis zu 188 Weiterleitungen am Tag. Mehrfach konnten über mehrere Stunden aufgrund der großen Anzahl an Hilfeersuchen in der Leitstelle der KV keine Anrufe über die gemeinsame Schnittstelle weitergeleitet werden und mussten hier im Ersatz mit Einsatzmitteln der Berliner Notfallrettung bearbeitet werden.

**Figure 2:**
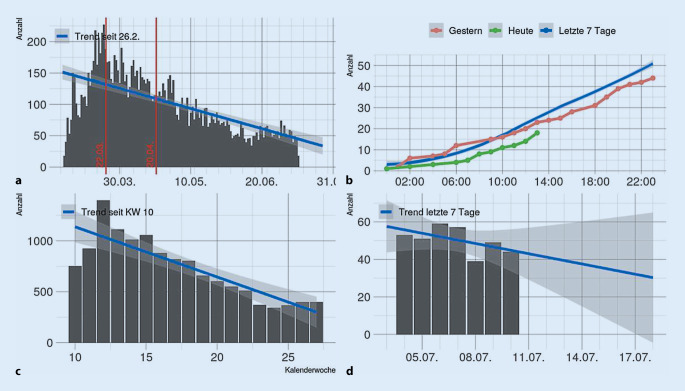

**Figure 3:**
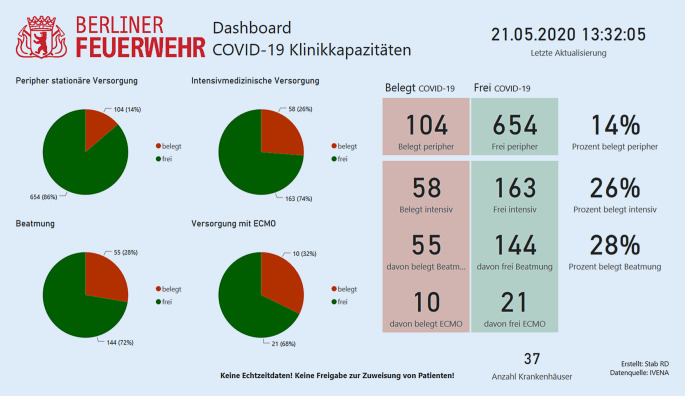

Mit dem Aufwachsen der epidemiologischen Lage wurde in der Berliner Feuerwehr ein „virtual operations support team“ (VOST) implementiert. Ziel dieses Teams war es, die Lagebeurteilung und Entscheidungsfindung durch IT-gestützte Analysen und Visualisierungen zu unterstützen. Eine weitere Aufgabe des VOST war die Entwicklung von Ad-hoc-IT-Lösungen, beispielsweise Miniapplikationen zur Lagerbestandsüberwachung oder für die Betreuung der Mitarbeiterhotline. Hierfür wurde ein Team von IT-Spezialist*Innen aus verschiedenen Abteilungen der Berliner Feuerwehr zusammengestellt und in die Struktur des Pandemiestabes integriert. Durch die Krisenstäbe der Senatsverwaltungen des Landes Berlin wurde dem Einsatzstab der Berliner Feuerwehr die Aufgabe der zentralen, organisationsübergreifenden digitalen Lagedarstellung für das Land Berlin übertragen. Aufgabe des VOST war deshalb die kontinuierliche Darstellung der wichtigsten „key performance indicators“ (KPI) in diversen Dashboards und digitalen Anwendungen. Technologisch im Zentrum standen dabei insbesondere das Geoinformationssystem ArcGIS der Fa. ESRI, Redlands, Kalifornien, USA, Office-365-Produkte der Fa. Microsoft, Redmond, Washington, USA, die App NaProt zur digitalen Einsatzberichtserstattung der Fa. Pulsation IT, Berlin, Deutschland und die Einsatzsteuerungssoftware FIRE der Fa. BananaGlue, Berlin, Deutschland. Durch die verschiedenen Anwendungen war es möglich, das der Pandemielage geschuldete spezifische Einsatzgeschehen (Abb. 2), die Systemauslastung, einschließlich Leitstellen, KPI (Bettenzahlen, Zuweisungen, Anrufaufkommen), Klinikkapazitäten (Abb. 3) sowie longitudinale Fallzahlentwicklung (Zusatzmaterial online: „Live-Daten zum longitudinalen COVID-19-Einsatzaufkommen in der FIRE APP“, s. Hinweis am Anfang des Artikels) und Live-Einsatzdaten (Zusatzmaterial online: „Live-Daten zu einem laufenden COVID-19-Einsatz in der FIRE APP auf dem iPad des Einsatzfahrzeugs“, s. Hinweis am Anfang des Artikels) zur Darstellung zu bringen.

Besondere Bedeutung zur Identifikation von potenziellen Hotspots, beispielsweise Ausbruchgeschehen in organisierten Wohnformen, hatte dabei das Software-Tool FIRE-Analytics. Einsatzdaten wurden statistisch analysiert und COVID-19-bezogene Einsätze in Pflegeeinrichtungen in Echtzeit zur Darstellung gebracht. So konnten den behördlichen und politischen Entscheidungsträgern ad hoc Informationen aufbereitet zur Verfügung gestellt werden (Zusatzmaterial online: „Live-Daten zum Einsatzaufkommen in Pflegeeinrichtungen in FIRE Analytics“, s. Hinweis am Anfang des Artikels).

## Bewältigung der Lage

Gemäß der Fahrzeug- und Funktionsverteilung werden in der Berliner Notfallrettung im Tagdienst planmäßig 351 Funktionen (Einsatzkräfte) auf 172 Fahrzeugen und 78 Rettungswachen besetzt. Krankentransporte sind entsprechend dem Gesetz über den Rettungsdienst für das Land Berlin (RDG) nicht Teil der Notfallrettung, sondern werden privatwirtschaftlich organisiert durchgeführt. Insgesamt sind an den Aufgaben der Berliner Notfallrettung über 4000 Einsatzkräfte (rettungsdienstliches Fachpersonal) von insgesamt 12 unterschiedlichen Organisationen sowie zusätzlich über 500 akkreditierte Notärzte aus einem der 38 Berliner Notfallkrankenhäuser beteiligt.

Kennzeichnend für den gesamten Zeitraum der Pandemie war die hohe Dynamik, mit der sich die Rahmenbedingungen, das Einsatzgeschehen selbst, sowie die verfügbaren Informationen und Ressourcen veränderten. Eine zentrale Aufgabe über den gesamten Zeitraum der Pandemie bestand deshalb in der internen Krisenkommunikation. Im Zentrum standen dabei die fortlaufende Aktualisierung von SOP sowie die Aktualisierung von einsatztaktisch relevanten Informationen. Weiterhin waren Schwerpunkte die Vermittlung aktueller Erkenntnisse zu COVID-19-Erkrankungen sowie die Bedeutung von Hygiene- und Schutzmaßnahmen.

Ein wichtiger Kanal der Informationsbündelung war darüber hinaus die tägliche Lagefortschreibung, in der zentral an alle Organisationen, Dienststellen und jede einzelne Einsatzkraft, der aktuelle Sachstand, wesentliche Neuerungen sowie die wichtigsten Informationen gebündelt per E‑Mail durch den Einsatzstab der Feuerwehr herausgegeben wurden. Durch eine offene, ehrliche Kommunikation sollten den Mitarbeitenden Ängste genommen, Vertrauen geschaffen und der Verbreitung von Falschinformationen vorgebeugt werden. Kondensiert wurden diese Informationen – soweit für den Einsatzbetrieb unmittelbar von großer Bedeutung – auch auf den Wach-Displays (Bildschirmen auf allen Wachen), über Wachdurchsagen oder direkt in den Einsatzfahrzeugen zur Wahrnehmung gebracht.

Neben den täglichen Lagefortschreibungen und den internen Bulletins des Stabes für Führungskräfte wurden die wichtigsten Entscheidungsträger täglich um 12 Uhr per Videokonferenz über das laufende Geschehen und erforderliche Entscheidungen ins Bild gesetzt. Für alle Fragen zum Thema Arbeitsschutz, Quarantänemaßnahmen und Umsetzung des Infektionsschutzgesetzes wurde eine rund um die Uhr erreichbare Hotline für alle Einsatzkräfte eingerichtet. Jenseits der internen Kommunikation wurde via *Twitter, Facebook* und *YouTube* die Öffentlichkeit über die Arbeit der Einsatzkräfte, aber auch alternative Ressourcen wie Sonderrufnummern oder allgemeine Verhaltenshinweisen informiert.

In der Berliner Notfallrettung sind alle medizinischen Handlungsanweisungen in SOP geregelt [8]. In der laufenden Pandemie wurden durch die ÄLRD Berlin 15 Sonder-SOP rund um den Umgang mit dem COVID-19-Ausbruch zusätzlich veröffentlicht (Tab. 2) und insgesamt 58-mal aktualisiert.

**Table 2:** 

Name	Erstveröffentlichung	Versionen	Inhalt
1.1 Einsatzablauf Notfallrettung	24.01.2020	24	Die erste SOP. Beschreibt den Einsatzablauf für alle in der Notfallrettung Beteiligten (siehe Abb. 4) mit allen Falldefinitionen und den daraus resultierenden Konsequenzen. Aufgrund der Fülle an Änderungen der RKI-Kriterien waren zeitweise tägliche Updates erforderlich
1.2 Behandlungs- und Zuweisungsstrategie	17.03.2020	5	Beschreibt die Zuweisungsstrategie für Patienten mit Verdacht auf COVID-19-Erkrankung unter Würdigung des Patientenzustands und in Abstimmung mit dem SAVE-Berlin-Konzept
1.3 Intensivverlegung	19.03.2020	3	Regelt den Ablauf von Intensivverlegungen von der Anmeldung und Planung bis zur Durchführung im Rahmen des SAVE-Berlin-Konzepts
1.4 Mitnahme von Begleitpersonen	24.03.2020	2	Änderung der Strategie zur Mitnahme von Begleitpersonen in Anbetracht der in den Kliniken geltenden Besuchsverbote und des möglichen Infektionsrisikos durch die Personen selbst
1.5 Transportabschlussdesinfektion	14.04.2020	3	Regelung der Transportabschlussdesinfektion nach bestätigten COVID-19-Fällen
1.6 Reanimation	17.04.2020	1	Schulungs-SOP zu Besonderheiten in der Reanimation im Kontext COVID-19 mit Hinweisen, beispielsweise zu Einsatzstellenmanagement, Umgang mit Ersthelfern oder Todesfällen
1.7 Risikobewertung	06.05.2020	2	Weitere Schulungs-SOP, die die „awareness“ der Einsatzkräfte gegenüber dem teilweise schwer vorhersehbarem, fulminanten Verlauf von COVID19 steigern soll. Mit Hinweisen zu „red flags“ und Risikofaktoren
2.1 Intubation	18.03.2020	4	Beschreibt Schritt für Schritt einen optimierten Ablauf für die Intubation mit Fokus darauf, eine Aerosolbildung so gering wie möglich zu halten
3.1 Einsatzablauf NotSan-Erkunder	02.04.2020	1	Beschreibt den Einsatzablauf des neu aufgestellten Einsatzmittels NotSan-Erkunder und die verschiedenen Handlungsoptionen am Einsatzort
3.2 Checkliste NotSan-Erkunder	02.04.2020	1	Eine Checkliste für den Einsatz der NotSan-Erkunder, die auch die Indikation für den Einsatz des Telenotarztes regelt
3.3 Dokumentation Telenotarzt	02.04.2020	2	Beschreibung der Dokumentation durch den Telenotarzt im Rahmen der gemeinsamen Einsätze mit den NotSan-Erkundern
3.4 Einsatzanlage Erkundung Organisierte Wohnformen	24.04.2020	1	Beschreibt die Möglichkeiten zur Identifikation von möglichen COVID-19-Hotspots in organisierten Wohnformen (z. B. Pflegeheimen)
3.5 Ablauf Erkundung Organisierte Wohnformen	24.04.2020	1	Einsatz-SOP, die den Ablauf einer Erkundung in organsierten Wohnformen regelt
4.1 Meldewege	27.03.2020	2	Eine „interne“ SOP, die sämtliche Meldewege für bestätigte Fälle oder Verdachtsfälle sowohl bei Patienten als auch bei Mitarbeitern beschreibt
4.2 Management von Kontaktpersonen	25.03.2020	6	Umfangreiche SOP, die ebenfalls für den internen Gebrauch an der Mitarbeiter-Hotline erstellt wurde. Darin werden die Identifikation und das Management von Kontaktpersonen aller Kategorien geregelt

**Figure 4:**
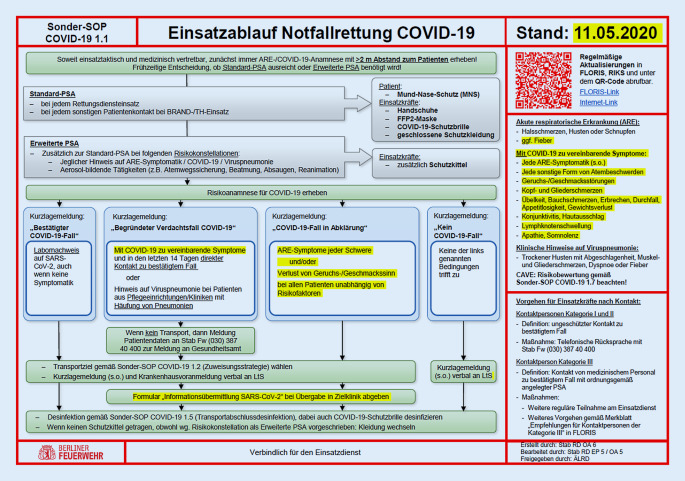

Wichtigsten Baustein für die Einsatzkräfte stellte dabei die „Sonder-SOP-COVID-19 Einsatzdienst“, die zentrale Handlungsanweisung für die Einsatzkräfte, dar (Abb. 4), in der neben Hinweisen zu Schutzmaßnahmen alle wichtigen Informationen zur Fallklassifizierung, zum Verhalten der Einsatzkräfte an der Einsatzstelle und notwendige, durchzuführende Maßnahmen geregelt waren. Alle SOP wurden jeweils mit der täglichen Lagefortschreibung und frei zugänglich im Internet (https://www.berliner-feuerwehr.de/service/mediathek/fachthemen/) veröffentlicht sowie auf den Wach-Displays und den iPads der Einsatzfahrzeuge zur Verfügung gestellt. Über einen QR-Code auf jeder Sonder-SOP konnte neben Versionsdatum durch die Anwendenden die Aktualität der vorliegenden Version überprüft werden.

Bereits in der Frühphase der pandemischen Ausbreitung von COVID-19 zeigte sich, dass es lageimmanent mehr und mehr zu Einschränkungen in der Regelversorgung kam. Während zunächst in zunehmender Anzahl Hausarztpraxen aufgrund von Personalausfall und fehlender Schutzausrüstung nicht mehr zur Verfügung standen [9], war auch die Versorgung durch den Ärztlichen Bereitschaftsdienst der Kassenärztlichen Vereinigung (ÄBD-KV) nicht mehr gewährleistet, da dieser im Land Berlin grundsätzlich über keine persönliche Schutzausrüstung (PSA) für den Einsatzdienst zur Versorgung infektiologischer Patienten verfügte [10]. Vor dem Hintergrund der wachsenden Zahl niedrigprioritärer Hilfeersuchen von Patienten mit ARE-Symptomen, die entsprechend der standardisierten Notrufabfrage eigentlich hätten an die KV-Leitstelle (116117) übergeben werden sollen, von dort aber in Ermangelung verfügbarer Fahrzeuge bzw. PSA wieder zur Leitstelle der Berliner Feuerwehr (112) zurückvermittelt wurden, entschied sich die Berliner Feuerwehr in Ersatzvornahme Teile des ÄBD in Zusammenarbeit mit und von der KV zu übernehmen [10]. Täglich wurden somit 4 sog. KV-ARE-Erkunder-Fahrzeuge durch die Berliner Feuerwehr in Betrieb genommen. Diese wurden von der Leitstelle der Berliner Feuerwehr disponiert und zu Einsätzen geschickt, die eigentlich durch den ÄBD hätten durchgeführt werden sollen. Die Fahrzeugführerfunktion wurde durch einen Mitarbeiter der Berliner Feuerwehr (Qualifikation mindestens Rettungssanitäter und langjährige Einsatzerfahrung in der Notfallrettung) gestellt; weiterhin wurde das Fahrzeug mit einem Arzt aus dem Personalpool des ÄBD der KV besetzt. Neben dem regulären Leistungsspektrum des ÄBD erfolgte zusätzlich die Ausstattung der KV-ARE-Erkunder-Teams mit PCR-Abstrich-Sets, sodass auch systematische Testungen beispielsweise bei Ausbruchgeschehen in Gemeinschaftsunterkünften durch diese Teams möglich waren [11]. Insgesamt konnten im Zeitraum März bis Juni 2020 so bei 1262 Einsätzen der KV-ARE-Erkunder 1585 Patienten versorgt werden, von denen bei 1265 ein Abstrich durchgeführt werden musste. Die durchschnittliche Versorgung am Einsatzort dauerte aufgrund der aufwendigen hygienischen Vorsichtsmaßnahmen sowie der erforderlichen Sorgfalt bei einer sehr heterogenen Beschwerdelage im Mittel 40 min. Zusätzlich zu den KV-ARE-Erkundern der Berliner Feuerwehr wurde durch die KV die hausärztliche Beratungsleistung in der Leitstelle der 116117 intensiviert, um – soweit fachlich möglich – Hilfeersuchen rein telefonisch abzuarbeiten.

**Figure 5:**
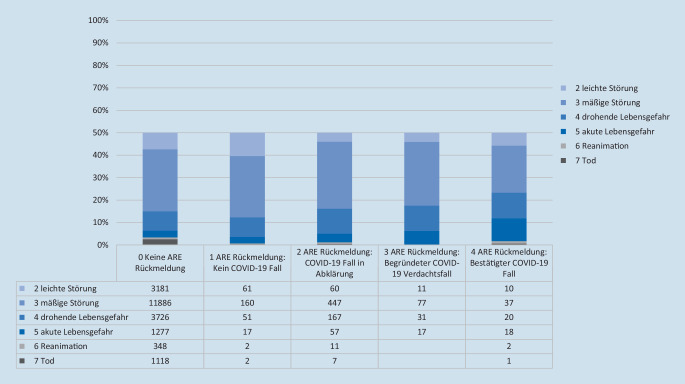

Als besondere Herausforderungen von COVID-19 zeigte sich insbesondere die teilweise schwierige telefonische Triagierung der an COVID-19 erkrankten Patienten, zum einen aufgrund der unspezifischen Symptomlage und zum anderen aufgrund der teilweisen rapiden Verschlechterung des Zustands einiger Patientinnen und Patienten innerhalb weniger Stunden. Vor diesem Hintergrund musste auch eine große Anzahl der Patienten mit moderater Krankheitslast zur differenzialdiagnostischen Entscheidungsfindung im Notarztdienst versorgt und überwiegend in ein Krankenhaus transportiert werden (Abb. 5). Um zu vermeiden, dass sämtliche Patienten bereits schon mit milden Symptomen in eine Klinik transportiert werden mussten, und zur Anbindung an alternative Versorgungsformen zum Krankenhaus wurden 4 weitere Einsatzfahrzeuge als „NotSan-Erkunder“ in Dienst genommen, die mit einem NotSan-Auszubildenden als Fahrer und einem besonders erfahrenen Notfallsanitäter der BFRA besetzt waren. Aufgabe der NotSan-Erkunder war es, innerhalb kurzer Zeit mit ihrem Fahrzeug Patienten aufzusuchen, deren Hilfeersuchen gemäß Notrufabfrage ansonsten, wenn auch weniger prioritär, mit einem RTW beschickt worden wären. Am Einsatzort wurde durch die NotSan-Erkunder eine systematische Untersuchung anhand einer standardisierten Checkliste durchgeführt. Bei kritischen Patienten oder bei Patienten mit einer stationären Behandlungsbedürftigkeit erfolgte die unmittelbare Nachalarmierung der erforderlichen Einsatzmittel (RTW und ggf. NEF); bei Patienten, deren Gesundheitszustand und Versorgungssituation eine ambulante Versorgung zuließen, erfolgten nach telefonischer Abstimmung mit dem Telenotarzt eine erweiterte Aufklärung und nach Möglichkeit die Entscheidung zu einer ambulanten Versorgung oder den Patienten an andere Versorgungsformen beispielsweise eine Follow-up-Visite durch eines der KV-Erkunder ARE-Teams zu vermitteln. Während der Hochphase der ersten Welle der Pandemie konnten im April 2020 bei 341 Einsätzen durch NotSan-Erkunder 49 % aller Patienten anstelle eines Krankenhauses an alternative Versorgungsangebote angebunden werden [12].

Neben der individuellen Patientenversorgung wurden das Einsatzmittel NotSan-Erkunder gemeinsam mit dem OAvD außerdem zu Ausbruchsgeschehen in organisierten Wohnformen zur Erkundung mit entsprechender Triagierung und Versorgung der Bewohner eingesetzt.

## Gewonnene Erkenntnisse

**Figure 6:**
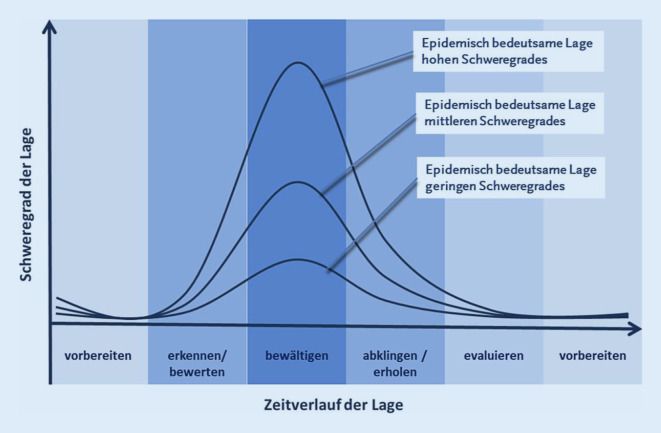

Grundsätzlich konnten bei der andauernden Lagebewältigung zu COVID-19 im Bereich der Notfallrettung im Land Berlin medizinische, organisatorische und technische sowie die verfügbaren Ressourcen betreffende Herausforderungen unterschieden werden (Tab. 3). Schweregrad und Zeitverlauf der COVID-19-Pandemie folgten dabei dem vom RKI beschriebenen typischen Verlauf von epidemiologisch bedeutsamen Lagen (Abb. 6, nach [13]).

**Table 3:** 

Medizinisch	*Wiederholt wechselnde Falldefinitionen/-klassifikationen* ⇒ permanentes Monitoring und Bewertung aller zur Verfügung stehenden wissenschaftlichen Fachinformationen zur Abwägung von Kontinuität und notwendiger Änderungen zugunsten erhaltender Kompatibilität durch mehrere zuständige Oberärzte der Ärztlichen Leitung des Rettungsdienst
	*Schneller Wandel hinsichtlich empfohlener Maßnahmen (Schutzmaßnahmen/Therapie)* ⇒ kontinuierliche, regionale Übersetzung des geltenden Standes der medizinischen Wissenschaft durch die Ärztliche Leitung des Rettungsdienstes
	*Schwierige Einschätzung der Erkrankungsschwere und Dynamik* ⇒ starke Ausweitung der überwiegend telefonischen Beratung und Supervision des Einsatzgeschehens durch den diensthabenden OAvD. Entwicklung eines digitalen Wächter-Tools bei wiederholten (ARE.-)Einsätzen zur gleichen Einsatzörtlichkeit
	*Schwieriges Monitoring von Risikogruppen, insbesondere organisierten Wohnformen/Obdachlosigkeit* ⇒ Entwicklung eines digitalen Analyse-Tools und automatisierten Reportings zu Anzahl und Häufungen von Einsätzen und Einsatzanlässen in Einrichtungen je Bewohner/Bett
Organisatorisch	*Große Dezentralität und Heterogenität im öffentlichen Gesundheitsdienst (ÖGD) und bei der Umsetzung des Infektionsschutzgesetzes (IFSG)* ⇒ mindestens wöchentliche, teilweise tägliche Telefon- oder Videokonferenzen mit allen regionalen Akteuren des Gesundheitswesens, insbesondere dem ÖGD
	*Begrenzte Leistungs- und Aufwuchsfähigkeit alternativer Versorgungsangebote (116117, Pflegedienste, psychiatrische Versorgungsangebote)* ⇒ bleibende Herausforderungen mit konsekutiver Ersatzvornahme der Berliner Notfallrettung überall dort, wo andere Strukturen oder Angebote nur noch eingeschränkt oder gar nicht mehr zur Verfügung standen
	*Unterschiedliche Ausstattung/Ausrüstung der beteiligten Organisationen der Notfallrettung* ⇒ Umstellung in den Mangelressourcen von einer dezentralen, organisationsbezogenen auf eine landesweite, gemeinsame Beschaffung aller am Gesundheitswesen beteiligten Akteure
	*Akzeptanz erforderlicher Hygienemaßnahmen und Schutzausrüstung in der Frühphase der Pandemie* ⇒ kontinuierliches Veränderungsmanagement durch Information, Schulung und wiederkehrende Sensibilisierung
Technisch	*Erreichungsgrad aller Einsatzkräfte bei hoher Informationsdichte und geringer Halbwertszeit* ⇒ Systematisierung und Konzentration der Kommunikationswege auf einige wenige kontinuierlich genutzter Kanäle nach dem Prinzip „low dose – high frequency“ (täglicher Lagebricht, Wachdurchsagen zum Schichtwechsel, Videobotschaften)
	*Umstellung aller regulären Besprechungsformate auf Videokonferenzen* ⇒ umfangreiche Investition in Videokonferenztechnik und Software-Lizenzen, Telefonweiterleitungskapazitäten, VPN-Technik
	*Umstellung aller Schulungskonzepte auf Webinare* ⇒ Entwicklung umfangreicher Blended-Learning-Curricula für bestehende Aus- und Fortbildungsformate
	*Wiederholtes Zusammenbrechen der 116117 wegen unzureichender Telekommunikationsinfrastruktur und konsekutivem Anstieg der Last auf der 112* ⇒ Ausbau der Leistungsfähigkeit der Telekommunikationsinfrastruktur bei der KV und zur Kompensation in der integrierten Leitstelle der Berliner Feuerwehr, einschließlich der Vorbereitung vollwertiger Redundanzen
Verfügbare Ressourcen	*Unzureichende Lagerbestände/Reserven bei Verbrauchsmaterialien (On-Demand-Versorgung)* ⇒ Vorratshaltung von 3 Monaten bei allen Verbrauchsmaterialien, einschließlich Medikamenten
	*Unzureichende Anzahl verfügbarer Schutzausrüstung* ⇒ umfangreicher Aufbau erheblicher Lagerbestände für Schutzausrüstung, gemessen am aktuellen Verbrauch
	*Fehlende Testmöglichkeiten und Laborverträge für Einsatzpersonal und Patienten* ⇒ Aufbau einer eigenen Teststrecke für das Einsatzpersonal in Zusammenarbeit mit der Polizei Berlin sowie für den Test von Patient*innen mit dem Labor Berlin
	*Starke Kompetition um insbesondere notärztliches Personal (Notärzte für ITW) mit den Kliniken, da dort derselbe Personenkreis für die Intensivstationen gebraucht wird* ⇒ Frühzeitige Kommunikation des Personalsonderbedarfs auf Basis flächendeckend bestehender Notarztverträge mit den Berliner Krankenhäusern; im Rahmen dessen auch, soweit möglich, Separieren unterschiedlicher Dienstgruppen zu Unterbrechung und Redundanz bei Infektionsketten

Insgesamt zeigten sich Herausforderungen im Zusammenhang mit COVID-19 insbesondere überall dort, wo auch im Alltag bereits Verbesserungspotenzial hinsichtlich Zuständigkeiten, Strukturen und Prozessen von den beteiligten Akteuren identifiziert war. Durch die besondere Tragweite, Intensität und Dynamik der epidemiologischen Entwicklungen kamen teilweise bekannte Schwächen besonders deutlich zum Tragen. Wenn gleichwohl kaum ein Bereich unserer Gesellschaft nicht unmittelbar und umfassend von den Auswirkungen von COVID-19 betroffen ist, so kann zweifellos festgestellt werden, dass die Notfallrettung als Teil der Gesundheits- und Sozialsysteme in Deutschland besonders stark und in erster Linie an der Kompensation und Bewältigung der Auswirkungen beteiligt ist. Gleichsam einem roten Faden wurde immer dann deutlich, dass die Notfallrettung „letzte Wiese“ der Versorgung und struktureller Verantwortung ist, wenn andere – primär zuständige – Versorgungsstrukturen versagten. Notfallrettung und Notfallmedizin bilden damit unterhalb der Katastrophe das entscheidende Rückgrat für die Resilienz unserer Gesellschaft und die Akzeptanz der Funktionsfähigkeit von unmittelbarer Daseinsführsorge. Notfallrettung geht in Letztzuständigkeit und Leistungsspektrum weit über eine Lotsenfunktion in der Leitstelle der 112 oder die Abwendung einer unmittelbaren Gefahr für Leib oder Leben hinaus. Über die Bewältigung der aktuellen Herausforderungen von COVID-19 hinaus wird es Gegenstand laufender Gesetzgebungsverfahren sein, dieser faktischen Rolle des erweiterten und ultimativen Sicherstellungsauftrags der Notfallrettung in einem qualitativ und quantitativ gewachsenen Aufgabenspektrum unserer Gesundheitssysteme Rechnung zu tragen [14].

Die gewonnenen Erkenntnisse in der Berliner Notfallrettung decken sich mit den Erfahrungen anderer internationaler Rettungsdienstorganisationen. So konnten Ventura et al. Herausforderungen durch einen erheblichen Mangel erforderlicher Schutzausrüstungen und bestehende Unsicherheiten im Umgang mit COVID-19-Patienten im Bereich verschiedener Rettungsdienste in den USA feststellen [15]. Fernandez et al. konnten zeigen, dass mit den dem Rettungsdienst zur Verfügung stehenden Mitteln eine sichere Identifikation von COVID-19-Patienten nur unzureichend möglich ist und deshalb z. B. hinsichtlich der Schutzbekleidung grundsätzlich bei allen Einsätzen von einem infektiologischen Risiko ausgegangen werden muss; dies deckt sich mit dem Vorgehen in den Berliner SOP [16]. Anders als Lai et al. für den Rettungsdienst in New York festgestellt haben, konnte während der andauernden COVID-19-Pandemie in der Berliner Notfallrettung kein Anstieg an Reanimationen registriert werden [17]. Auch der von Lerner et al. in den USA beschriebene, zwischenzeitlich starke Rückgang der Einsatzzahlen des Rettungsdienstes ist in Berlin deutlich flacher ausgefallen [18] und entspricht eher dem von Hagebusch et al. für die Stadt Frankfurt a. M. beschriebenen Umfang anderer deutscher Großstädte [19]. Sechi et al. hingegen beschrieben, dass, wie in Berlin auch, in der Lombardei die Einführung von „business intelligence (BI) tools“ in der Leitstelle und im Rettungsdienst entscheidend zur Bewältigung der Herausforderungen der Pandemie beigetragen hat [20]. Auch die von Sayers et al. geforderte Nutzung von aggregierten Daten des Rettungsdienstes für (gesundheitspolitische) Entscheidungsträger konnte mit dem VOST-Team der Berliner Feuerwehr bereits umgesetzt werden [21]. Die Bedeutung der Einführung von ambulanten COVID-19-Testkapazitäten des Rettungsdienstes zur Vermeidung unnötiger Krankenhausvorstellung vulnerabler Patientengruppen, so wie sie in Berlin umgesetzt wurde, deckt sich ferner mit den durch Goldberg et al. für den Rettungsdienst in Boston beschriebenen Erfahrungen [22].

## Fazit für die Praxis

- COVID-19 stellt eine außerordentliche Belastungsprobe für das Gesundheitswesen insgesamt und die notfallmedizinische Versorgung im Besonderen dar.
- Die Notfallrettung übernimmt die entscheidende Rolle der ambulanten Notfallversorgung und präklinischen Patientensteuerung insbesondere auch dort, wo andere Versorgungsangebote nicht oder nur noch eingeschränkt zur Verfügung stehen.
- Die Bewältigung von epidemiologisch bedeutsamen Lagen in der Notfallmedizin erfordert eine frühzeitige, systematische Lageüberwachung, eine detaillierte, digitale Lagedarstellung und eine agile, an regionale Gegebenheiten angepasste Lagebewältigung.
- Neue Einsatzmittel insbesondere im Bereich niedrig prioritärer Hilfeersuchen können helfen, den besonderen Herausforderungen einer Pandemie besser zu begegnen.
- Die hohe Dynamik epidemiologischer Entwicklungen erfordert kontinuierliche, systematische Kommunikation und stets aktualisierte, praktische, medizinische Handlungsempfehlungen (SOP).

## Caption Electronic Supplementary Material




