# Supplementary material for: COVID-19 Stress test for ensuring emergency healthcare: strategy and response of emergency medical services in Berlin
Source: Anaesthesist. 2020 Nov 27;70(5):420–31. [Article in German] doi: 10.1007/s00101-020-00890-8 (PMC7692437; doi:10.1007/s00101-020-00890-8)

Zusatzmaterial zum Beitrag

„COVID-19 – Stresstest für die Sicherstellung der Notfallversorgung: Strategie und Maßnahmen der Notfallrettung in Berlin“

Janosch Dahmen[1 2 3], Linnart Bäker[1], Florian Breuer[1 2], Karsten Homrighausen[1], Christopher Pommerenke[1], Jan-Karl Stiepak[1 2], Stefan Poloczek[1 2],
[1] Berliner Feuerwehr
[2] Ärztliche Leitung Rettungsdienst im Land Berlin
[3] Universität Witten/Herdecke, Fakultät für Gesundheit, Department Humanmedizin

Publikation in *Der Anaesthesist* 2020

Der dazugehörige Beitrag und das Zusatzmaterial stehen Ihnen auf www.springermedizin.de zur Verfügung. Bitte geben Sie dort den Beitragstitel in die Suche ein.

Falldefinitionen und Kurzlagemeldungen in Analogie zur RKI-Falldefinition

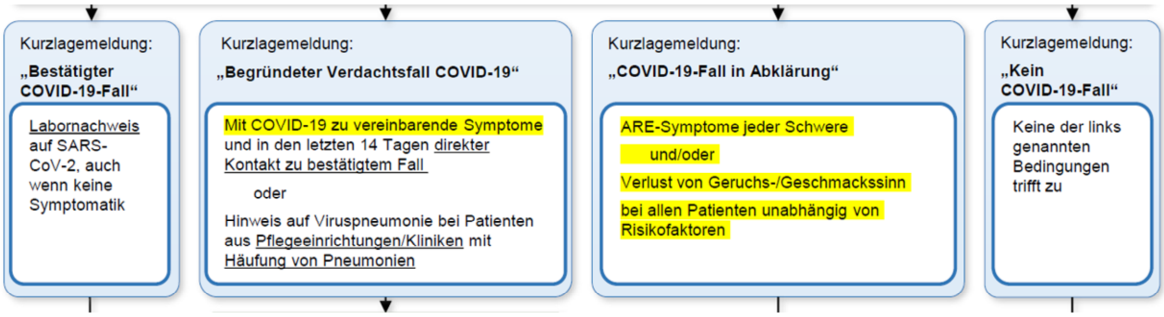

Live-Daten zum longitudinalen COVID-19 Einsatzaufkommen in der FIRE APP

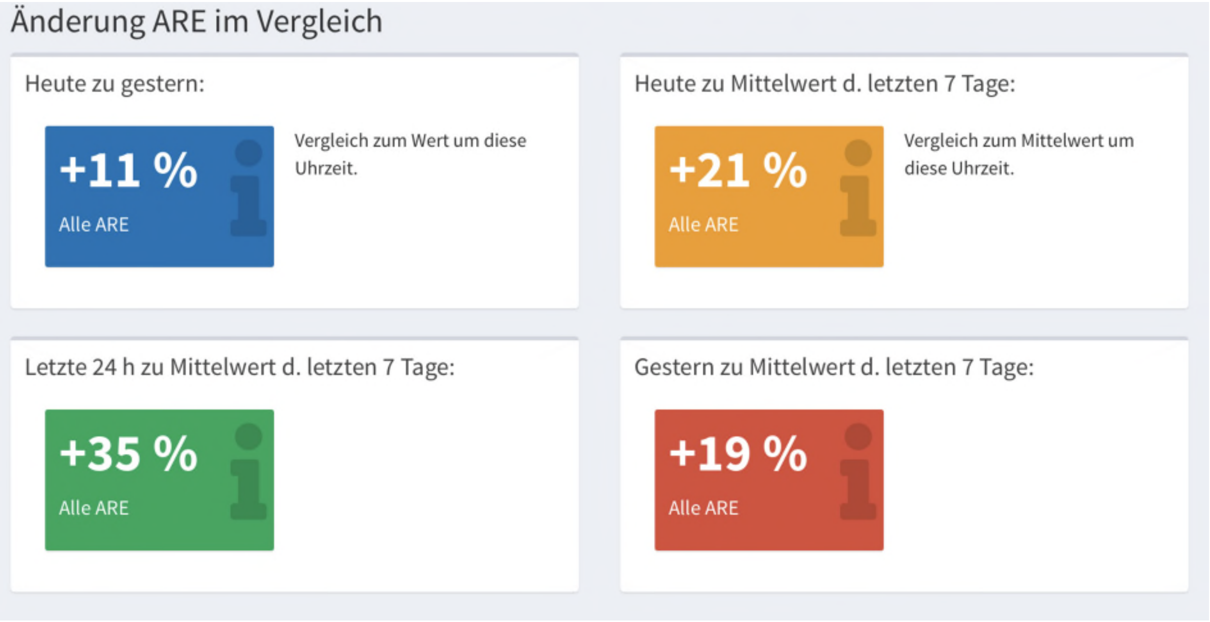

LIVE-DATEN ZU EINEM LAUFENDEN COVID-19 EINSATZ IN DER FIRE APP AUF DEM IPAD DES EINSATZFAHRZEUG

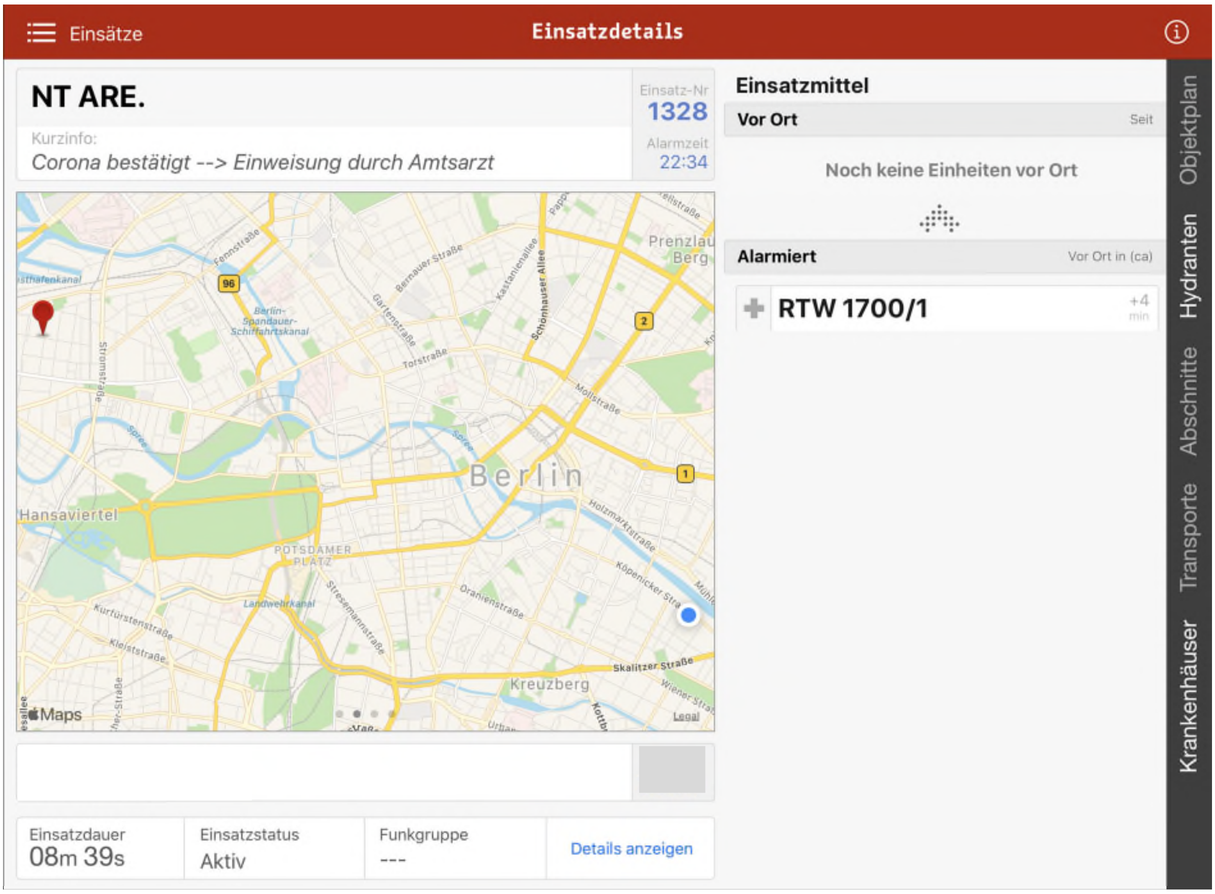

LIVE-DATEN ZUM EINSATZAUFKOMMEN IN PFLEGEEINRICHTUNGEN IN FIRE ANALYTICS

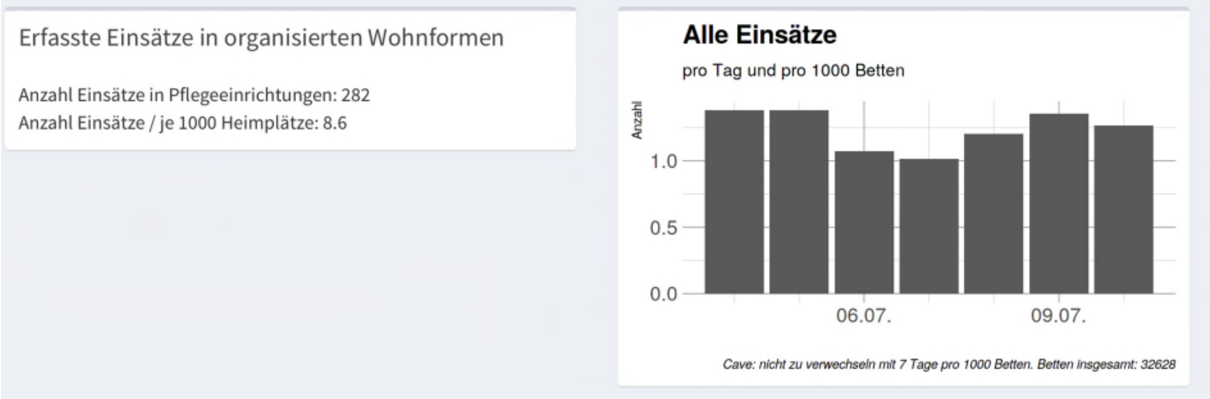

Supplement: Supplementary file 1 [file 101_2020_890_MOESM1_ESM.pdf]
